# A method for the detection and characterization of technology fronts: Analysis of the dynamics of technological change in 3D printing technology

**DOI:** 10.1371/journal.pone.0210441

**Published:** 2019-01-07

**Authors:** Gaizka Garechana, Rosa Río-Belver, Iñaki Bildosola, Ernesto Cilleruelo-Carrasco

**Affiliations:** Department of Business Management, University of the Basque Country (UPV/EHU), Bilbao, Spain; Institut Català de Paleoecologia Humana i Evolució Social (IPHES), SPAIN

## Abstract

This paper presents a method for the identification of the “technology fronts”—core technological solutions—underlying a certain broad technology, and the characterization of their change dynamics. We propose an approach based on the Latent Dirichlet Allocation (LDA) model combined with patent data analysis and text mining techniques for the identification and dynamic characterization of the main fronts where actual technological solutions are put into practice. 3D printing technology has been selected to put our method into practice for its market emergence and multidisciplinarity. The results show two highly relevant and specialized fronts strongly related with mechanical design that evolve gradually, in our opinion acting as enabling technologies. On the other side, we detected three fronts undergoing significant changes, namely layer-by-layer multimaterial manufacturing, data processing and stereolithograpy techniques. Laser and electron-beam based technologies take shape in the latter years and show signs of becoming enabling technologies in the future. The technology fronts and data revealed by our method have been convincing to experts and coincident with many technology trends already pointed out in technical reports and scientific literature.

## Introduction

Decision making in science and technology (S&T) is an uncertainty-plagued process that combines the expertise and information internally available at the firm with the thorough analysis of external variables that exert influence on the rate/direction of the evolution of technology. The set of techniques and information sources that are put into practice with this purpose, among others, form a well-developed academic and managerial discipline called Future-oriented Technology Analysis (FTA) [1], which spans several activities such as technology foresight, forecasting and technology roadmapping [2], all of which share the purpose of optimizing decision making in S&T. Most of FTA combines eclectic quantitative and qualitative information sources, patent data usually being a frequent choice among the latter. This paper presents a method for the identification of the “technology fronts” underlying a certain technology, and the characterization of their change dynamics. We propose an approach based on the Latent Dirichlet Allocation (LDA) model combined with patent data analysis and text mining techniques for the identification and dynamic characterization of the main fronts where actual technological solutions are put into practice.

What we understand by “technology front” could be described as follows: “core technological solutions underlying certain device or broad development”. The improvement of complex devices over time is often shaped by the incorporation/adaptation of new elements on the device: for example, a new material is used, key features of design are changed, new sensors are embedded and new control systems are put into practice. A good case can be found in the automotive industry: the incorporation of more than 4km of electric wire brought entirely new features to cars and increased their efficiency by gradually substituting previous mechanical/hydraulic solutions, and wireless data transmission might shape the next generation of devices to solve certain shortcomings of current automotive electric wiring-based systems [3]. In this work we aim to detect and characterize the main fronts of technological change that characterize the evolution of a machine type that itself contains many devices that are evolving themselves. What really makes a technological innovation unique, thus patentable, have to be clearly stated in the patent claims: “patents claims are the heart of a patent (…) the claims demarcate in words the boundary of invention (…) only the technology covered in the claims is protected” [4]. For patents protecting a new 3D printer design, or any subset of components (such as a particular feeder-heater-nozzle design), the claims should state the points where technology has really changed with respect to prior art, and this is the textual field we propose to analyze in this work to detect and characterize the “technology fronts” underlying evolution in 3D printing technology.

**Fig 1:**
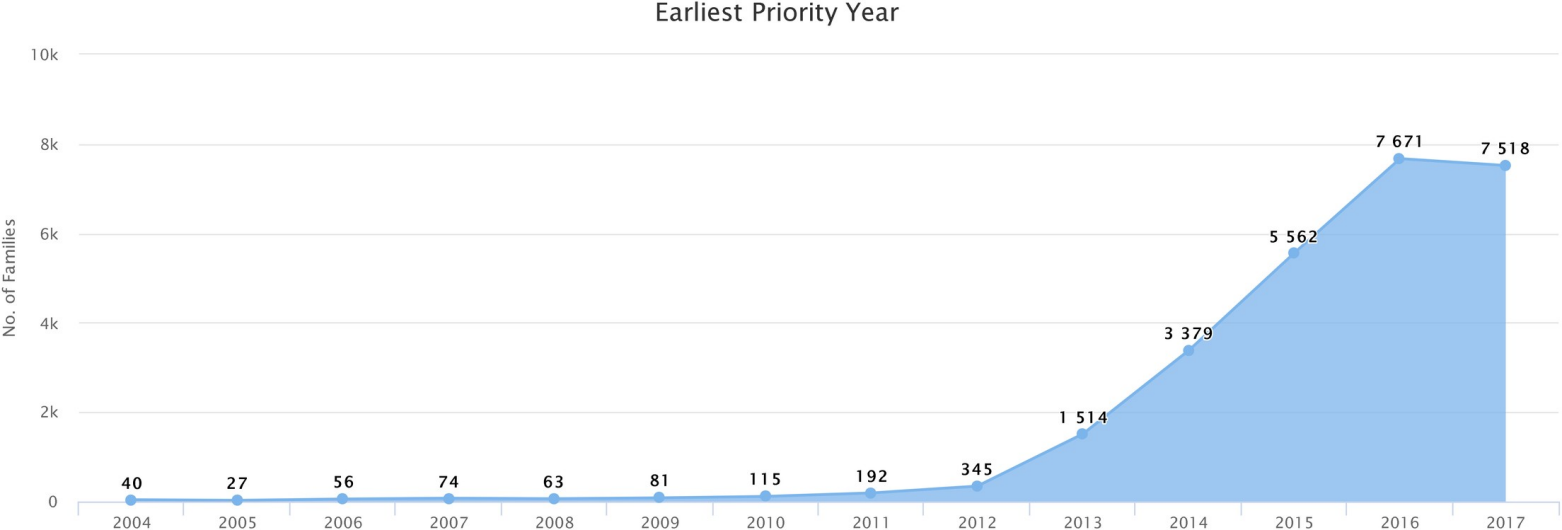
Evolution in the annual number of 3D printing technology patents (simple families) filed since year 2004. Screenshot of the number of patents filed each year since year 2004, retrieved by running the query presented in the “data download” section on the Patseer database (query run on 19 November 2018). The years on the x-axis correspond to the earliest priority year of the simple patent family. It should be noted that data corresponding to year 2017 might present a substantial amount of upside variation due to database updates.

3D printing technology is experiencing an explosion in the number of patents filed since 2013, according to the data retrieved using the query described in the “data download” section. The number of simple patent families filed that year more than quadrupled the number of patents filed in 2012, and the average growth rate in the number of patents filed each year from 2013 to 2016 stands at a remarkable 75%, as shown by Fig 1. Several authors [5,6] suggest that increasing sales, industry’s growing adoption and a boost in popularity have qualified 3D printing technology as “emergent”, notwithstanding, the first functional solutions in 3D printing date back 30 years.

Many authors trace the end of such “sleeping beauty” behavior back to the expiration of a set of patents protecting key 3D printing technologies, the development of certain enabling technologies and the high expectations on the possibilities of mass customization [7–10]. The corporate strategy of patent holders and the limitations of the early machines in terms of affordability, materials and printing quality are among other factors that could explain this phenomenon. The current position of 3D printing technology in the classic technology life cycle stages of emergence (or “introduction”), growth, maturity and saturation depends on the approach chosen in order to define the boundaries of each phase in patent filing analyses. According to Haupt [11], a strongly increasing number of annual patent filings characterizes the “growth” stage, a situation where technology and market uncertainties have disappeared and incremental innovations take the lead in the evolution of the technology: The “emergence” stage is characterized by a slowly growing upward trend, and according to our data 3D printing would have gone beyond this stage in year 2013. At this point it should be noted that an increase in patents typically precedes the introduction of a new technology in the market [12], so 3D printing technology can be considered an emerging technology when analyzing other indicators such as product sales or adoption by industry. Other authors consider that the emergence stage is characterized by a substantial increase in patent activity after a period characterized by a stable number of yearly patents [13,14], using this approach, 3D printing technology could be considered an emerging technology that has just started its growth phase.

However, the influence of patent expiration on new developments in 3D printing [6–9] may reoccur in the coming years, due to the recent expiration of certain patents on basic technologies in this field [15]. This would confirm our hypothesis that this technology is at the beginning of a growth phase, possibly pointing at a rise in scientific, public and entrepreneurial attention to 3D printing technology for the years to come. This combination of emergence and multidisciplinarity makes 3D printing a fairly heterogeneous field where many techniques and applications evolve at different paces, yet sharing the same broad technological concept, i.e., building an object by adding the material(s) that form that object on a layer-by-layer basis, thus forming a common technological principle. These are a few of the facts that led us to choose 3D printing to put our method into practice.

Another reason for choosing this field to conduct our study is the following: 3D printers are devices formed by a wide array of mechanical and electronic components that aim at solving technological challenges such as the processing of information for 3D layer-by-layer manufacturing, the multimaterial processing capabilities, rheology problems, high precision positioning technologies, adherence between layers, etc., thus being fertile in “technology fronts” to be detected, that is to say, key points where technology improvements are taking place, as explained in the paragraphs above. Last but not least, our research team is linked with firms and maker communities in 3D printing that deliver the necessary expert assessment for technology-specific analyses.

### 3D printing technology

The disruption that 3D printing technology is expected to bring is bound to transform business models from the dependence of economies of scale and the massive outsourcing of production facilities to a less wasteful, logistically far more efficient approach, based on mass customization and the re-location of manufacturing centers near the main markets where sales actually take place, thus giving a new boost to the principles of Just in Time production of goods. In addition to this, the manufacturing of complex geometries would be cost-efficient almost regardless of the manufacturing batch size, and materials wasted would be negligible when compared to traditional subtractive manufacturing methods. A special socio-economical challenge posed by the arrival of this technology will be the destabilizing effect of the deep transformation—or sheer reduction—that traditional manufacturing labor force will have to endure under this new paradigm [16,17]. Some authors even draw parallels between the irruption of the mp3 file format and the diffusion of Internet and the widespread adoption of 3D printing technology, suggesting that the days of patent and other copyright protection systems may be numbered [18].

In this section we will provide an overview of the main technologies that currently form the state-of-art in 3D printing, using the standard terminology for additive manufacturing technologies defined by committees ISO/TC 261 and F42 from ISO and ASTM, respectively, and published under the standard ISO/ASTM 52900:2015 [19]. This overview aims at giving a bird eye’s view of the techniques forming the 3D printing field, more technical details about the techniques described here can be found in the excellent review written by Ngo et al. [20].

#### VAT photopolymerization

**Fig 2:**
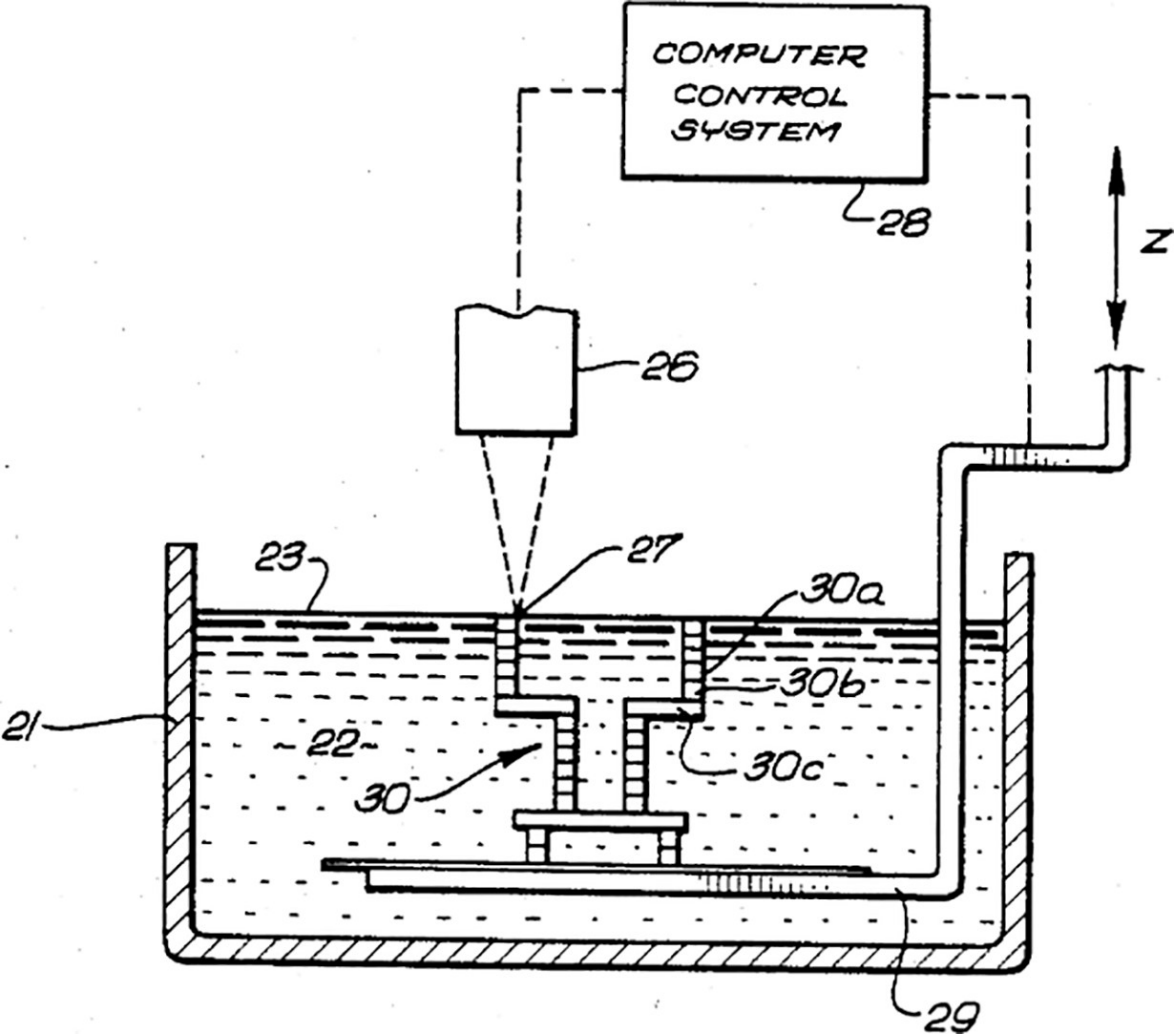
Sketch corresponding to the first VAT photopolymerization machine. Original sketch of the “apparatus for production of three-dimensional objects by stereolithography”, extracted from the US patent US4575330A.

Photopolymers are a particular type of liquid polymers that polymerize (we could say solidify or harden, for 3D printing purposes) when exposed to visible or ultraviolet (UV) wavelengths. The most frequent commercial materials used in this technology are acrylates, epoxies and vinyl ethers, and well-known applications of photopolymerization include the plastic coating of paper or cardboard and tooth fillings using dental composite. The first patent of a 3D printing machine based on VAT photopolymerization, titled “Apparatus for production of three-dimensional objects by stereolithography” was filed in 1984 by Charles Hull, hence the popularity of the name “stereolithography” to refer to this technology [21]. The typical design of a 3D printing machine based on this technology is formed by a platform that controls the Z axis and a light source that can be directed to solidify the polymer in the desired points on a layer-by-layer basis. The platform will move downwards as the printing progresses, and once the product is finished the remaining polymer liquid in the vat is evacuated. The original sketch (Fig 2) presented in the patent filed by Charles Hull is a good example of the basic design of such devices.

Additional features of these devices often include a passing blade system to improve the union between layers. In some cases an extra curing of the finished product is necessary for it to achieve the desired mechanical properties [22]. This is one of the 3D printing methods where the highest printing resolution can be achieved and a very promising technology for the field of bioengineering, where fully customized implants can be built in an efficient manner [23] as well as medical templates and biomodels for surgery preparation [24,25].

#### Material jetting

This technology follows a process similar to the conventional ink jet printers, in fact, it is frequently found in the literature under the heading of “inkjet printing”. Liquid materials with a varying degree of viscosity are deposited on a platform where they are hardened, either by drying, cooling or chemical reaction (this is the case of concrete 3D printers, for example [26]) or by curing with UV light. Most current industrial material jetting printers use piezoelectric drop-on-demand (DOD) systems, instead of continuous flow systems [27]. Material jetting is the main technique for 3D printing using ceramic materials in solution or colloidal form [20], the fabrication of geometrically complex bone-implants using compatible biomaterials is one of the applications gaining traction in this technology [28,29]

#### Binder jetting

**Fig 3:**
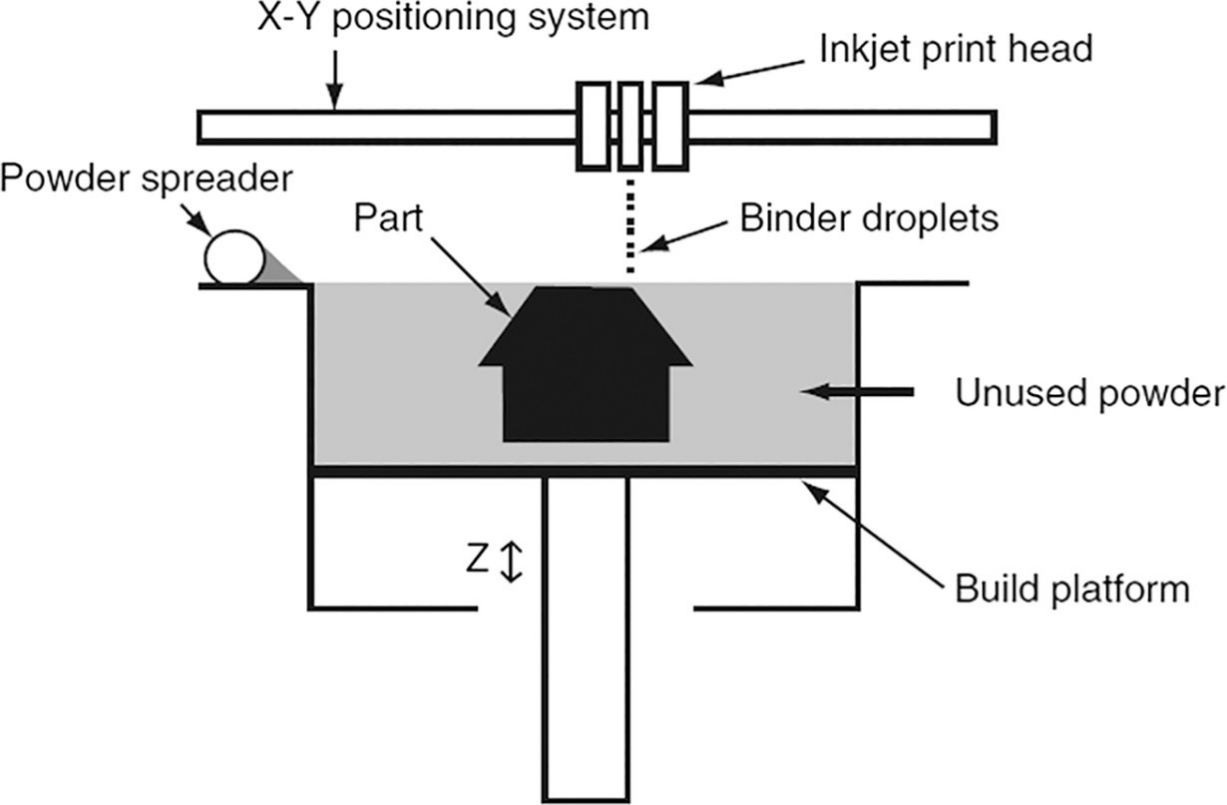
Binder jetting system. This figure represents the binder jetting system method, where a binder is deposited on a powdered material to create compact layers that give shape to the finished product. Source: [30].

This technology is often named three-dimensional printing or 3DP, and works by jetting a binding material on a certain area of a layer of powdered material, thus gluing together the base material and forming a compact layer. The machine then deposits a new layer of powdered material and the printing head deposits the adhesive material on the points corresponding to the next layer, these steps follow each other until the product is finished. Binder jetting devices usually have a mobile base platform (see Fig 3) in charge of determining the Z- axis position of the layer being printed. This method permits a greater range of materials, including polymers, ceramics and metals, and is also suitable for fabricating multi-material products that combine different materials on each layer, however, these advantages are offset by the fact that it often produces poorer precision and surface finishes than material jetting, and the resulting product is more porous, which decreases its mechanical properties. The fragility of the printed parts can be reduced by further finishing operations such as infiltration [30].

Binder jetting is the most successful 3D printing technique in the pharmaceutical industry, making it possible to customize key variables in drugs, such as release-characteristics, dosages and drug combinations, among others [31,32]. Operation at room temperature also makes this technique very suitable for building complete biostructures that embed both biological agents and living cells [33].

#### Material extrusion

**Fig 4:**
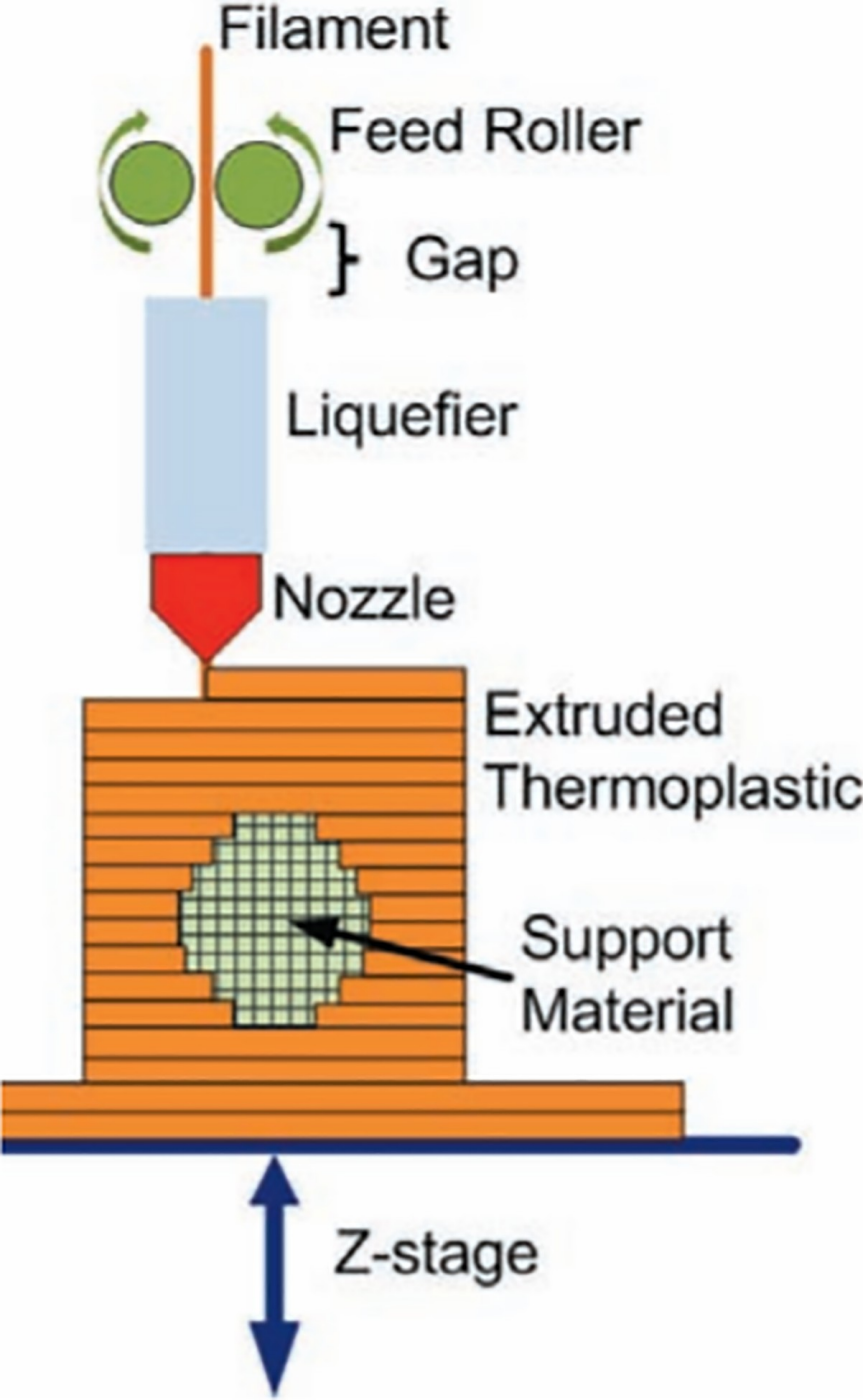
FFF printing system. Diagram showing the basis of the FFF technique. Source: [34].

This process shares some technical similarities with the material jetting technique but with the key difference of operating through a heated nozzle that merges and extrudes a material that is typically fed in a filament (solid) format, as shown in Fig 4. This technique is also known by the names “fused deposition modeling” (FDM) or “fused filament fabrication” (FFF) and was first patented by Scott Crump in 1989 (US Grant US5340433A) and commercialized by the firm Stratasys.

This is the most widespread and inexpensive (at least at basic level) process for 3D printing, and after the main patents that protected the technology expired, a vibrant open-design community has been grouped in the RepRap project, an initiative aimed at creating an affordable desktop manufacturing system that would enable the individual to self-manufacture a wide range of devices [35]. At a basic level this technique is inexpensive, but the accuracy and density of the manufactured products are usually below the levels that can be achieved with other 3D printing processes, even though the competitiveness of FDM is improving rapidly [36]. The thermoplastics Acrylonitrile Butadiene Styrene (ABS) and Polylactide Acid (PLA) are the most common materials used in FDM, however, the simplicity and affordability of this technique begs the development of new materials that would expand its application domain beyond the thermoplastics. Several studies have been published on the mechanical properties of ABS composites using both metallic and non-metallic elements, typically reporting poorer mechanical properties but improved thermal conductivity, dielectric permittivity and radiation shielding features in some metal-ABS composites [37,38]. The possibility of manufacturing sintered ceramic and metallic parts has also been studied; in this case the feedstock for the FDM process is usually formed by powdered ceramic/metallic material bound together in an organic matrix. After the part is printed, the organic binder is removed and sintering and/or infiltration treatments are used to finish the part [39]. The use of biomaterials in FDM printing techniques is mainly aimed at building biological scaffolds, commonly using polycaprolactone (PCL) and bioactive glass composites to build a structure that acts as an interface for facilitating the regeneration of cellular tissues. The majority of applications rule out directly incorporating living cells or biological agents to the printing material due to the high temperatures during the extrusion process [33].

#### Powder bed fusion

**Fig 5:**
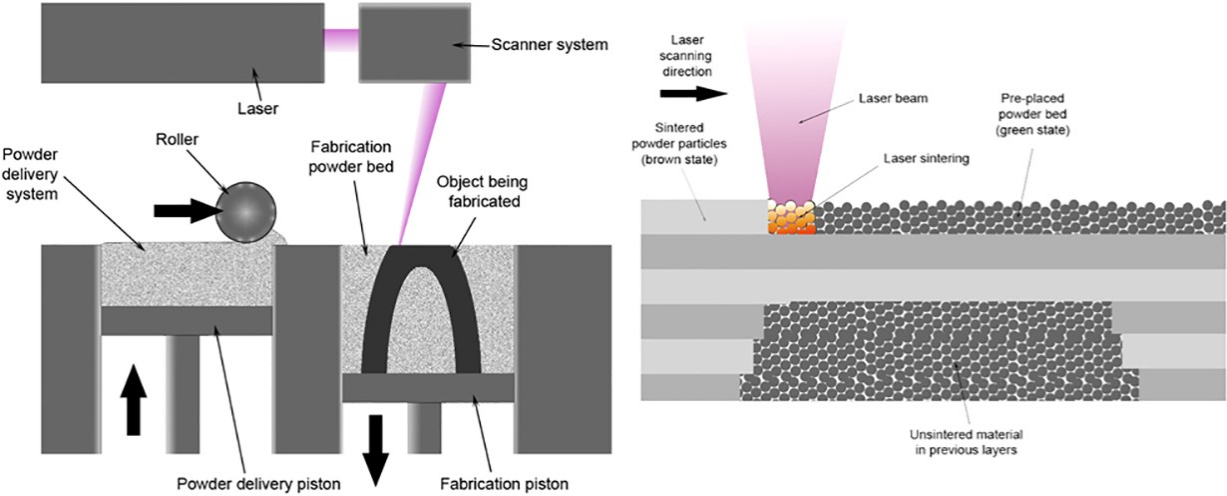
Selective laser sintering process. Schematic representation of the selective laser sintering process. Source: Materialgeeza /Wikimedia commons.

Several techniques that share common elements are grouped under this category: All powder bed fusion processes work on a powdered layer of the feedstock that will be selectively melted or sintered, on a layer-by-layer basis, until the finished product is formed, and blades or rollers (see Fig 5) are generally used for distributing the powder once the previous layer has been finished. Gibson [40] distinguishes between the laser-based and the electron beam-based techniques: Electron beam based techniques can only be used with metals, since the processed material must be conductive, while laser based techniques, in addition to metals, can also be used with ceramics and polymers. Electron beam techniques offer a more energy-efficient process but trail laser techniques in resolution and surface finishing quality.

Laser techniques are often divided into selective laser sintering (SLS) and selective laser melting (SLM) techniques, depending on the degree of melting achieved on the powder particles. Most commercial processes can be classified as “liquid phase sintering–partial melting” (corresponding to the most typical SLS methods) and “full melting” (corresponding to SLM) categories, but depending on the binding mechanism “solid state sintering” and “chemically induced binding” can also be defined [41]. Powder bed fusion techniques have been successfully used to produce metallic components with mechanical properties comparable to those manufactured by traditional means, however, steep temperature gradients and high cooling rates in the printing process can substantially alter the properties of the printed product [42]. The processing of biomaterials using SLS techniques has also produced highly satisfactory results, particularly in applications requiring high product resolution [43]. The range of metallic and ceramic materials that can be used as a feedstock for powder bed fusion techniques is highly dependent on the melting point, particularly in SLM techniques, which are based on the complete fusion of the materials being processed [20].

#### Sheet lamination

Sheet lamination differs from the rest of additive manufacturing techniques explained in this section in the fact that the feedstock is provided to the process in the shape of fully finished sheets that must be joined together. The most basic technology for sheet lamination is the laminated object manufacturing (LOM) technique, which directly uses adhesive materials or simple mechanical means to join the material sheets together. This technique includes several sub-classifications, depending on whether the material layers are feed to the process in their final shape or not (“bond then form” vs “form then bond”) [44]. Ultrasonic additive manufacturing (UAM) is used for metallic material sheet lamination, and is based on the sequential bonding of metal foils using ultrasonic metal welding, often requiring further mechanical processing of the welded foils by CNC milling [20]. Ultrasonic consolidation is also a promising technique for achieving the embedding of composite materials in a metal matrix, avoiding some of the disadvantages of melting and casting metals [45,46].

#### Directed energy deposition

The directed energy deposition technique uses a laser or electron beam in order to melt a material and shape a part on a layer-by-layer basis. The key difference between this method and the powder bed fusion method lies in the fact that in this case the material is not pre-laid, but is simultaneously deposited and melted on a surface, thus no powder bed is required. This method can be used with polymers, ceramic and metallic materials as long as it is based on a laser device, given that electron beam based devices can only work with conductive materials. The feedstock can be fed in powdered or wire form, wire is more convenient to store and process, but processing conditions are decisive to achieve the desired quality, with wire tip position, feed-rate and direction, laser spot size and laser power being some of the variables with higher impact on the resulting quality of the process. The applications based on powder are more frequent partly due to greater possibilities for using powdered additive materials [47].

**Fig 6:**
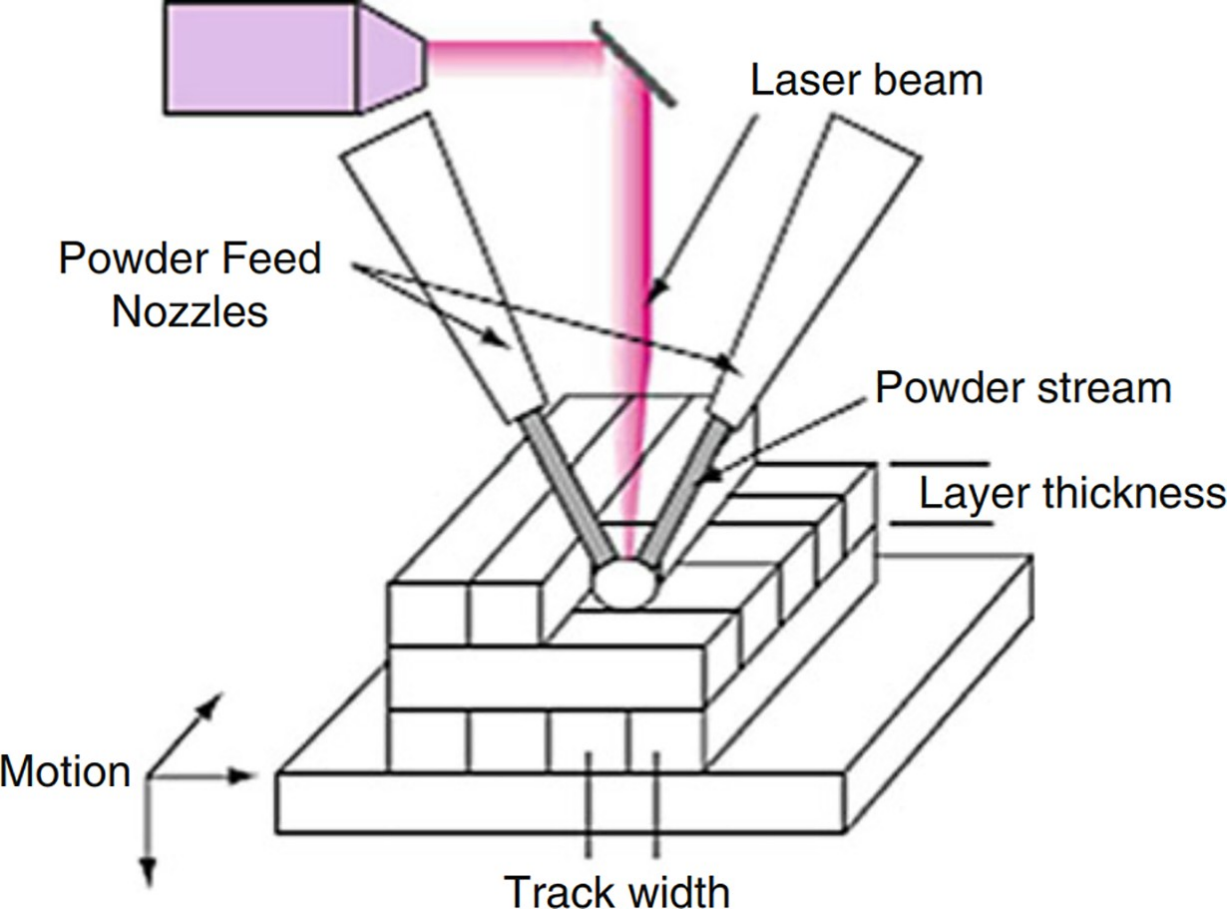
Sketch of a multiple nozzle direct energy deposition process. Schematic representation of a 2-nozzle, powder-based direct energy deposition process. Source: [48].

The processing material can be any metal powder that is weldable [49], and the feeding system can be coaxial to the laser or side-fed by using one or several nozzles, as shown in Fig 6. This technique is suitable for producing finished parts, but one of its key advantages lies in the possibility of printing on elements that are already built, thus many directed energy deposition applications are focused on restoration or cladding [20,50]

#### Barriers and improvement points

Despite already being a “hot topic” in the world of manufacturing technologies, according to a study published by PricewaterhouseCoopers [51] actual real-world implementations of 3D printing technology in manufacturing industries are predominantly (48.8%) located in prototyping and experimental facilities, while 13.2% of surveyed firms use this technology for both prototyping and production purposes and only 9.1% of firms use 3D Printing solely for manufacturing. The areas presented in Table 1 are considered future improvements that should occur in 3D printing in order to seize the multiple opportunities that this technology has to offer [52].

**Table 1:** Key points to improve in current state-of-art 3D printing [52].

AREA	KEY POINTS
Performance	Increased printing speed. Increased resolution. Possibility for autonomous operation.
Multi-material printing	Incorporation of multiple materials in the same object, including composites combining plastic and metallic materials [8].
Finished products	Ability to print fully functional and active systems that incorporate many modules, such as embedded sensors, electronics, etc.
Ease of use	Suppress sources of error and reliability failures, such as support structure generation, part orientation, auto-calibration.
Software	Ease of use for design & operation Optimization for accuracy Generation of printing files directly from existing objects or 2D images

This table explains some of the trends with higher influence on the developments in 3D printing.

When non-adopters were asked about 3D printing technology, the most cited reasons for not entering additive manufacturing were the cost of printers (42.1%) and the lack of internal expertise to fully exploit this technology (32.2%), followed by the uncertainty about the quality of printed parts (33,1%) and the slowness of printing process (25.6%). It is worth noting that the limited amount of materials suitable for 3D printing scores in fifth place (22.3%) [51]. The nature of these problems affects many parts and processes in the 3D printer and shows the transversality of the solutions that will be required. While both printers and printing materials are subject to limits based on physics, software improvements are set to introduce transversal developments that could accelerate the path to extensive implementation of 3D printing technology in many applications. Geometrically complex, multi-material product printing is a specific case where software solutions can add the ultimate edge to already available high-precision printing techniques [53], in addition to many successful applications of 3D printing to medical fields, thanks to the development of software solutions able to generate 3D objects directly from data obtained by tomography or magnetic resonance [54].

On the new material development side, there are also significant research fronts dealing with the development of new materials for 3D printing, particularly in metals. Metal printing is in many cases restricted to aluminum and brass in desktop printers, due to the high melting points of industrial-grade metals such as high tensile steel. Some authors state that this limitation could be overcome in the future by using nanoscale-sized metal particles, which fuse at dramatically lower temperatures as the particle size becomes smaller than 50 nm [52]. Improvements in the optical properties of the powder used (e.g., absorption, reflection and transmittance) are also decisive for achieving this purpose [55].

### Technology analysis

Technological knowledge is a vital support for strategy, innovation and operational processes at firms. The analysis of technology is underpinning management of technology both at micro (firms) and macro (R&D policies) levels, the information contained in patents being one of the crucial information inputs of such systems, both due to the wealth of information they contain and the availability of data and methods to extract activable information for pertinent decision making. The analysis of patent portfolios can also give relevant insight into the R&D goals pursued by competitors [56,57].

Understanding of the dynamics of technological change and the forecasting of future changes is another interesting output of patent analysis. Benson and Magee [58] proposed a method for estimating the rate of technological progress in a particular domain, using patent data. Those domains with higher a progress rate are deemed—at least temporarily—to dominate the competitive markets except for a few resistant niches, this evidences the potential of quantitative technology analysis for understanding the future of technology [59]. The different stages (emergence, growth, maturity and saturation) of the technology life cycle can also be identified using patent data, as shown by Gao et al. [60].

While many relevant S&T questions can be addressed by exploiting the structured data present on patents, there is a vast wealth of information available therein in the form of textual, unstructured information. Text-mining techniques allow the structuration, cleaning and further processing of semi-structured and unstructured textual data in order to make them suitable for feeding “conventional” data-mining procedures that would enable the extraction of relevant knowledge from data [61]. Natural Language Processing (NLP), term consolidation, thesaurization and noise-removal techniques are among the most frequent steps in text mining processes applied to textual information contained in abstracts, titles or claims of patents [62,63]. The review conducted by Abbas et al. [64] is recommended reading for an extensive description of the main types and purposes of text-mining analysis applied to patent data.

## Methodology

### Data download

The first step of this study required the building of a dataset containing the patents related to 3D printing technology. Initially, the approach of downloading the full B33 subclass “additive manufacturing technology” present in the IPC classification scheme was considered, however, a large number of patents simply describing objects that could be 3D printed fall into this category, thus not being descriptive of the 3D printing technological developments we were looking for. A deeper look into the subclass description shows that “this subclass is for obligatory supplementary classification of subject matter already classified as such in other classification places, when the subject matter contains an aspect of additive manufacturing”, thus confirming the presence of undesired records in this approach. The conventional method of building a query was put into practice instead, leading to the following query, adapted to the syntax of Patseer patent database [65]. The time limits were set in accordance with the filing of the first—one may even say foundational—patents in 3D printing technology [10]:

TA:((threew0dimens*w0print*)OR(3Dprint*)OR(additivew0manuf*))ANDPRD:([1985‑01‑01TO2017‑12‑31])

These terms were looked for in patent’s title and abstract fields, retrieving 22034 simple families of patents (from this point forward we will interchangeably use the term “patents”, referring to simple families of patents as returned by Patseer database) that were first filed (priority date) between years 1985 and 2017. This query was run on days 22, 23 and 24 of February 2018. The distribution of patents across the years was highly uneven; very few patents in 3D printing were filed in the 80’s and 90’s in contrast with the boom that took place from 2013 onwards. A review of the earliest patents led us to conclude that technology development was relatively stagnant during that period and that probably no significant information is lost by aggregating the patents corresponding to early years. Table 2 shows the time intervals that were set for studying the evolution of technology fronts across time:

**Table 2:** Time intervals set for the analysis.

YEARS	NUMBER OF PATENT FAMILIES
2017	2987
2016	6558
2015	5827
2014	3659
2013	1623
2006–12	1000
1985–2005	380

This table shows the time intervals set for this study and the number of patent families corresponding to each interval

As previously explained, patents contain vital information items for addressing several issues concerning technology evolution. The very core of the patent in the event of litigation or prosecution lies in its claims: these are textual sentences defining critical elements of the patent and usually the primary subject of examination. It seems reasonable to posit that patent claims could be the sentences that convey more information about the key aspects where the proposed technological solution adds value, and an appropriate information field for identifying the technology fronts underlying 3D printing, by means of text-mining.

### Text mining procedure

The dataset built in the previous step contains key information about the technology fronts underlying 3D printing techniques, but it is necessary to separate the wheat from the chaff in order to detect the main components or areas in which technological advance has occurred. Topic modeling is a machine learning technique that deals with the problem of automatically classifying sets of documents into themes, where each of the documents under study consists of a mixture of topics. In addition to this, each document has a “gamma” value for each topic, that could be interpreted as the proportion of words from each document that are generated from that topic [66], which notably improves the subsequent process of interpretation and labeling of topics [62,67]. Topic modeling has led to satisfactory results in automatic classification of scientific publications [68] and the robustness and limitations of the method for several types of data have also been tested [69]. The R package “topicmodels” was used in this study for fitting a Latent Dirichlet Allocation (LDA) model using the Variational Expectation-Maximization (VEM) algorithm [70].

**Fig 7:**
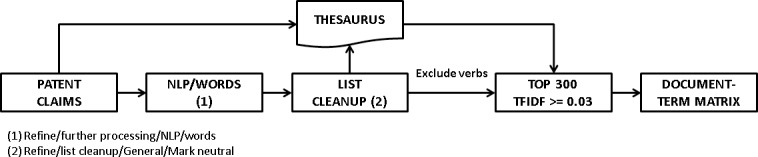
Text mining process. Diagram showing the text-mining steps leading to obtaining the document-term matrices. This process is replicated for each time interval.

The input for the LDA analysis is a document-term matrix containing the number of times each term occurs in the claims of each patent. Claims are a free text field, so a thorough text-mining cleaning process must be conducted in order to remove pronouns, adjectives and common-use terms such as “invention” that, being predominant in term frequency, are useless for interpreting the topic structure of data. Our text-mining process is described in Fig 7.

A first step consists in transforming patent claims using a proprietary NLP/Words algorithm in version 10.0 of Vantage Point (VP) text mining software [71] for sentence splitting and part-of-speech (POS) tagging. This step produces a list formed by the relative frequency of words and the POS identification of each word (noun, adjective, adverb, verb), while removing pronouns and other textual noise. However, from a semantic point of view the same word could be present more than once in this list due to word inflections, so the next step consisted in a cleanup in order to merge the inflected forms; this was done using the “list cleanup” command in VP, this process generates a thesaurus that contains the information of the inflected terms that have been merged into a single standard form. This same thesaurus has been used to standardize the terms present in the full text of patent claims. At this point we decided to exclude the verbs from the analysis, since verbs strongly need the presence of the adjacent words for their interpretation—this is the case of “comprise” or “move”, for example—while nouns, adjectives and adverbs, indicating material types, properties or machine parts have a more straightforward interpretation. This bag-of-words (BOW) approach inevitably involves the loss of the information contained in the syntactical structure of text, a loss that can be partially reduced by using higher order n-grams or complementing the BOW by using proximity indicators that capture the information contained in the syntactic order of the words present in text. However, the option of using N-grams in the subsequent steps was discarded because of the sparsity of resulting document-term matrices, which produced meaningless results.

The last step of the process consisted of a Term Frequency—Inverse Document Frequency (TFIDF) analysis, this process weighs a set of terms present in a collection of documents (patent claims, in this case), penalizing the terms that occur in many documents (these have a likelihood of being general terms, with no particular relevance to characterize the contents of a document) and giving advantage to those terms that occur frequently, but are not widespread in the document collection [72,73]. We pragmatically set a minimum TFIDF threshold of 0.03 to discard the “general” terms and took the top 300 terms—according to the number of patents where that word exceeded such a threshold—to build the document-term matrix with which to feed the LDA analysis. This process was replicated for each time interval described in section “data download”.

The authors recommend using the R packages “openNLP” [74] and “koRpus” [75] for reproducing the text mining steps that in this study have been conducted using VP proprietary software.

### Topic analysis and characterization

**Fig 8:**
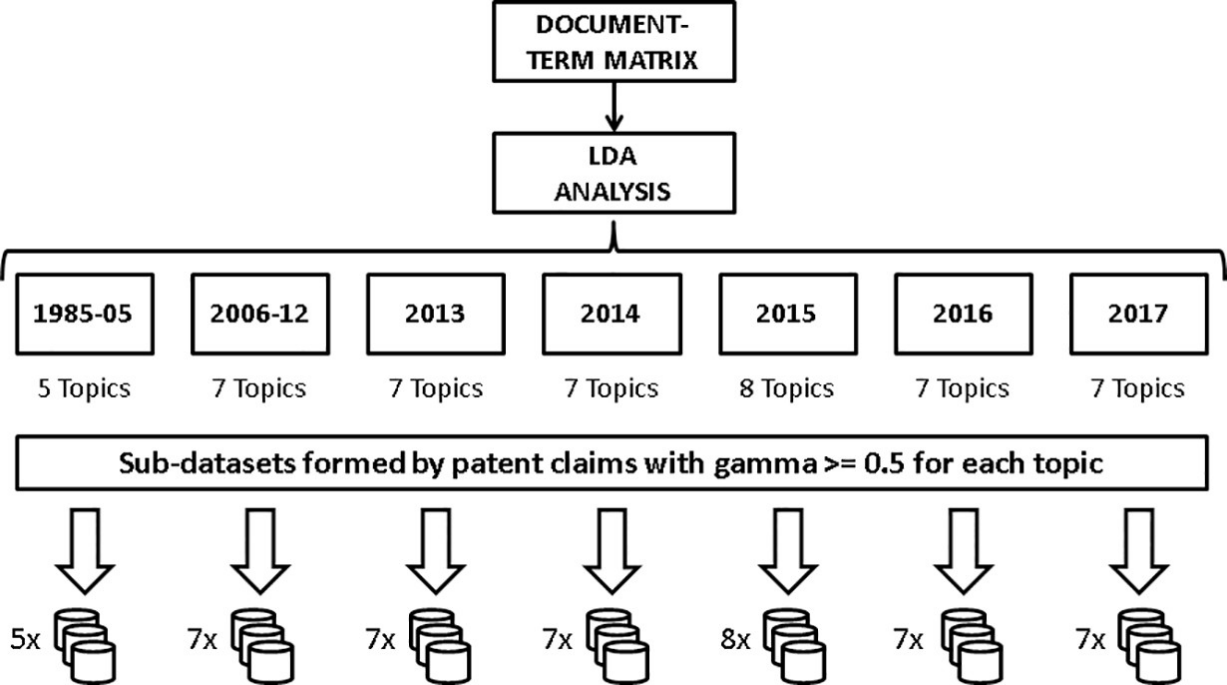
Topic modeling and sub-dataset building process. Diagram showing the process followed from the document-term matrices obtained by text mining to the building of sub-datasets containing the core information to characterize the technology fronts. The number of topics obtained for each time interval is shown.

The application of the LDA model requires setting the desired number of topics in advance. The output of the LDA showed differences between time intervals, partly due to the different sample size of each interval (see the reference Tang et al. [69]) nonetheless we detected that the more straightforward interpretation of topics was achieved using 7 topics for most of the intervals (see Fig 8). The interpretation of the topics was conducted combining the analysis of the terms and patents that had the higher beta/gamma values (top beta word list for words, gamma higher than 0.5 for patents), on each topic. As explained in “text mining procedure” section, the R package “topicmodels” produces an estimation of the likelihood of words (beta) and documents (gamma) being generated in each topic. Fig 8 describes this process and shows the number of topics that were set for LDA analysis in each time interval.

At this point of the analysis we are interested in characterizing the behavior of the technology fronts detected by using topic modeling. While some technology fronts can be reasonably adjusted to the quandaries of a certain technique in 3D printing (see results section) we expect to find transversal fronts that refer to technical elements that are present in a variable proportion across several patents which may not have these transversal elements as a core contribution. In this paper we propose a data subsetting method based on the gamma coefficients returned by the model, so we can build samples of patents in which claims have the higher probability of focusing on the technical element we wish to characterize. After interpreting the topics in each interval, we built several sub-datasets (one per topic and time interval) consisting of the patents that had a gamma coefficient equal or higher than 0.5 on each topic. According to our method, these datasets condense the patents that more clearly represent the essence of each technology front. Hereafter we will use the expressions “technology front” and “topic” indistinctly.

### Technology change characterization

A few topics had ambiguous interpretation and were discarded from this analysis, and seven clearly defined topics were identified that had a remarkable continuity across the analyzed time intervals, thus allowing the analysis of technological change over time. As previously explained in the “topic analysis and characterization” section, we built several databases for each time interval, each of the databases containing the patents that had a gamma factor higher than 0.5 on a given topic. We propose the following set of indicators based on patent data in order to study the evolution of technology fronts over time, the word in brackets is the abbreviation we chose for each indicator:

- Number of forward citations per patent (FORPAT): It can be stated that a certain correlation exists between the citations a patent receives and its technological impact on a particular field [59,76,77], or even on the economic value of the patent [78].
- Average age of backward citations (YEARBACK): Domains that cite more immediate patents should have higher rates of technological progress [79], as demonstrated by Magee & Benson [59].
- Patent international classification codes (IPCPAT, IPCNEW): Patent classification analysis can be used to detect important aspects of technological evolution such as the interdisciplinarity, which could lead to improved technological outcomes [59,77,79,80]. We propose an approach based on the analysis of the amount of unique IPC codes (at group level) per patent present on each technology front (IPCPAT) and the % of new IPC codes emerging for each time interval (IPCNEW). To qualify as “new IPC”, each of the IPC’s is tested against the cumulative IPC’s corresponding to previous years, so technologies with longer trajectories will have a significantly lower IPCNEW value than recently emerged technology fronts. A more accurate perception about the object of study change dynamics is achieved by comparing the technologies at the same point of their trajectories.

**Fig 9:**
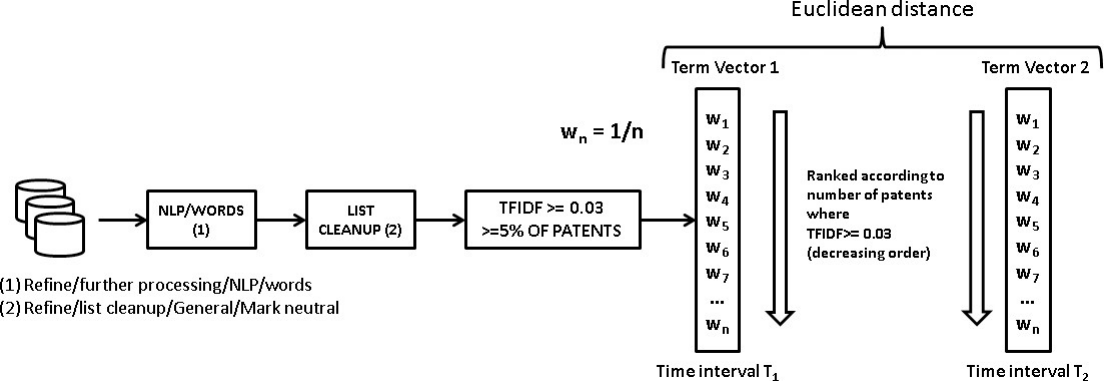
Process for building the term vectors. The information contained in the sub-datasets built in the previous step is text-mined in order to extract the key concepts therein and term vectors are built for each time interval, which will allow us to conduct a comparative analysis across time.

We complement these indicators with an additional text-mining study of technology changes. With this purpose in mind, we replicated the text-mining steps explained in the “text mining procedure” section on each of the sub-datasets, in order to identify the key terms that returned a TFIDF value higher or equal to 0.03 in at least one patent. In addition to this, the terms that were not present in at least 5% of the patents of the sub-dataset were removed. The terms in this list were arranged according to the number of patents where they got a TFIDF higher or equal to 0.03, in decreasing order, so the most relevant and significant terms were at the top of that list. Comparing these lists across time for each technology front could give us a measure of the rate of technology change therein. This was done by building a vector term were the terms were weighed according to the inverse value of their position in the list described above. This process is outlined in Fig 9.

These term vectors were built for each technology front and time interval, thereby allowing us to detect changes in the relevance of technological concepts over time. Terms that change their rank in the term vector from interval T1 to T2 will see their weight (1/n) changed. Terms that disappear from interval T1 to T2 (they do not exceed the TFIDF > = 0.03 and relative 5% presence threshold) get zero weight in the term vector corresponding to T2. This method gives a quantitative proxy of the technology change taking place in each topic by calculating the Euclidean distance between vectors corresponding to consecutive time intervals (TERMVEC). TERMVEC is a proxy to detect changes in the composition and relevance of the top concepts dominating a technological area. Moreover, we add another indicator to measure the % of unprecedented terms (terms occurring for the first time on a technology front) corresponding to each time interval. Only the terms that exceed the TFIDF and relative presence threshold are considered, so these concepts satisfy the dual condition of relevance and novelty (NEWTERM). A persistent and higher than average presence of these new terms is a signal of technology change over time [81]. Given that terms from each time interval are compared with the cumulative amount of terms present in previous intervals, comparisons between technology fronts should be made at the same point of maturity. Technologies generally have a higher chance of having many new terms in their early years of development.

It should be noted that the analysis of each technology front using these indicators is performed on a relative basis with respect to the values produced by the rest of the fronts. To be persistently above or below the average value will be the determining factor to analyze the influence of each variable on the rate of change of a given technology front.

## Results & discussion

The first feature concerning technology evolution in the 3D printing field to draw our attention was the evolution in total patent number shown in Table 2. The annual patent filings have been growing at an average rate of 65% from year 2013 to 2016, with this rate dramatically dropping in year 2017: that year added just under half of the patents added the year before. A re-run of our query as of 14 May 2018 (Patseer database is updated weekly with new additions/modifications of contents) confirms this slowdown but moderates the drop, 2017 patent filings fall 37% when compared to 2016 data. According to plain patent production, there is enough evidence to consider that 3D printing may be achieving maturity in some of its main technologies.

**Fig 10:**
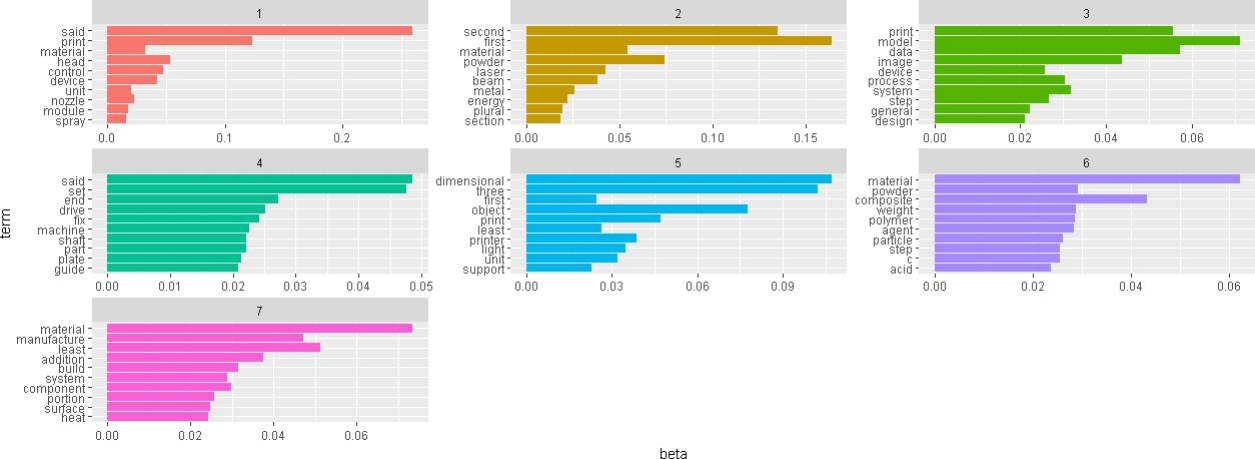
Topic structure corresponding to year 2014 data. 7-topic solution produced by LDA model with data corresponding to year 2014.

The LDA model has produced coherent results by setting 7 topics for most of the time intervals analyzed. As explained in the “methodology” section, topic interpretation is considerably eased by the beta/gamma values associated with every word and patent in the dataset, which reflect the probabilities of such words or patents being generated from that topic. Fig 10 shows the 7-topic structure corresponding to data from year 2014. The terms with higher probability (beta) of being generated in each of the topics are listed.

**Fig 11:**
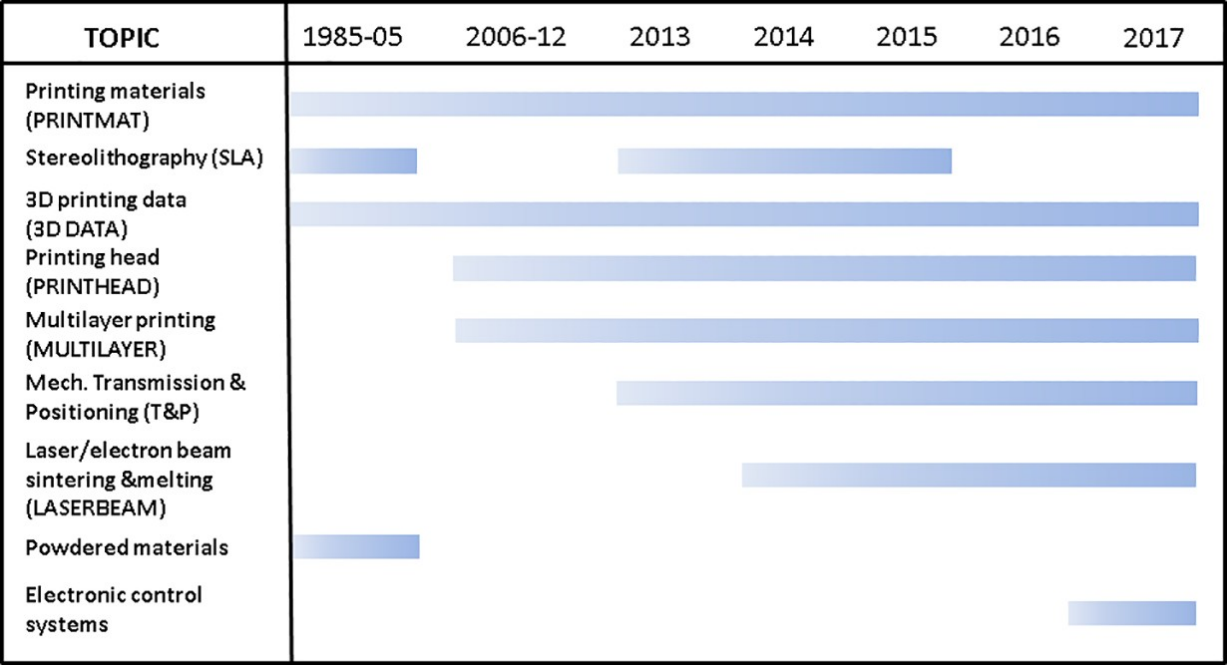
List of technology fronts. Labels of the technology fronts detected in the sub-datasets and the time span each technology front is present.

We found an interesting pattern in data that at the same time enabled us to conduct a technology change analysis: there is a stable set of technology fronts (topics) that can be detected in several consecutive time intervals. Fig 11 shows the full list of identified technology fronts and the time span each of them is present.

The following is a brief description of each front, according to our analysis of the terms and patents with highest beta/gamma on each. The names in brackets are the abbreviations for each front:

- Printing materials (PRINTMAT): Patents on new materials for 3D printing, including composites and mixtures of elements, with a description of physicochemical properties.
- Stereolithography (SLA): Devices involving a full array of photo-curing technologies, its raw materials (mainly polymeric resins), apparatus design, light sources and methods for obtaining products using this technology.
- 3D printing data (3D DATA): Data obtaining, processing and data input for 3D printing purposes. Automatic obtaining of printing data from 3D models or 2D images. Scanning. Data transfer systems.
- Printing head (PRINTHEAD): Patents heavily related with printing head configurations, temperature, feed and other control-related issues and designs, including multi-nozzle and anti-obstruction designs.
- Multilayer printing (MULTILAYER): Printing methods involving multi-layer technology, mainly either involving printing with different materials or making layers—surface treatments included—on a preexisting, not necessarily 3D printed material.
- Mechanic transmission & positioning (T&P): Patents dealing with precision positioning of parts/printing head, step-by-step motor elements and the mechanic transmissions used in 3D printing technology.
- Laser/electron beam melting & sintering (LASERBEAM): Both melting and sintering-based 3D printers (and the parts thereof) aimed at metal/composite material printing. Both laser and electron beam based devices are present.
- Powdered materials: A topic present only in 1985–2005 year interval, mainly dealing with powder-based 3D printing techniques with a noticeable presence of medicine-release systems.
- Electronic control systems: User-machine interaction devices and systems for automatic control of various aspects of 3D printing machines or networks formed by them. Topic present only in year 2017.

Experts confirm the coherency of the technology front structure present in our data, corroborating that topic modeling has unveiled both main technologies behind 3D printing and critical elements of these devices that strongly influence the success of developments in this industry. As explained in the “methodology” section, our next step consisted in building sub-databases for each topic, containing the set of patents that had a gamma higher or equal to 0.5. This data subsetting allowed us to conduct a highly-focused technology change analysis on each topic. Technology fronts “Powdered materials” and “Electronic control systems” were excluded from this part of the analysis due to lack of observations, so subsequent steps of the study refer to the remaining 7 fronts that show certain continuity. Another fact that should be noted is the absence of the SLA front in some of the time intervals. After considering its removal, we opted for including it in the technology analysis for the following reasons: First, the topic is very straightforwardly interpreted; our data clearly shows that this is a neatly defined technology front in 3D printing. Second, we think that it is advisable to leave room for a certain amount of variability when using dimensionality-reduction or other heuristic techniques on time series: temporal gaps should be allowed and the focus should instead be placed on the broader picture.

**Fig 12:**
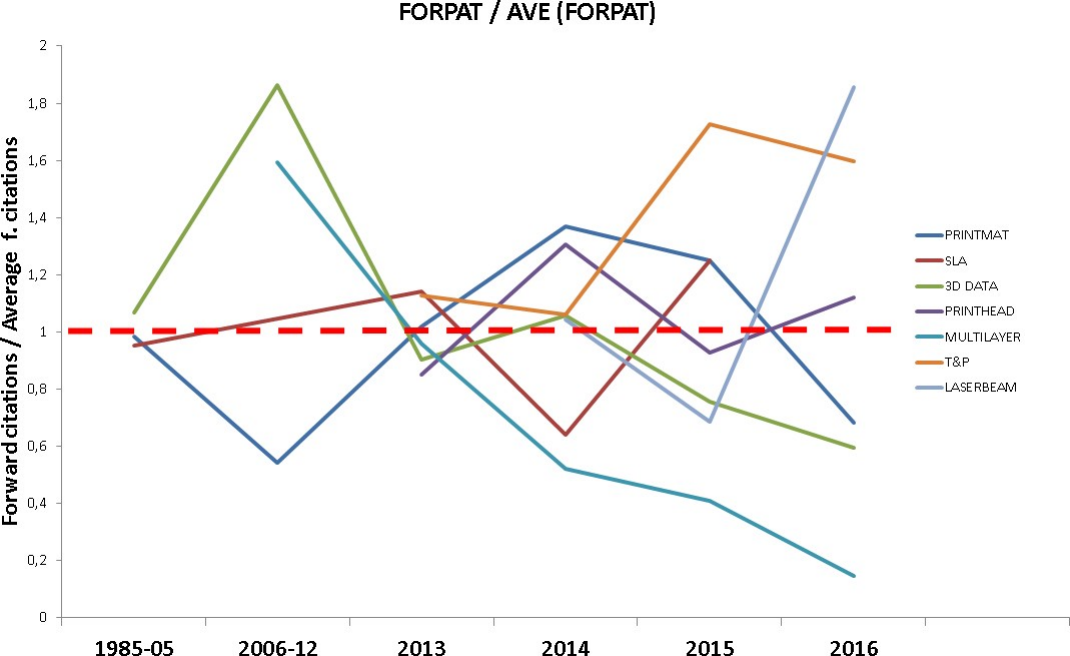
FORPAT for each technology front. Evolution of the FORPAT indicator for each technology front, normalized. Points above the red dashed line indicate higher than average forward citations.

The analysis of the forward citations per patent on each dataset (FORPAT) shows a downward trend across the entire interval analyzed, for all the technologies: this was something to be expected since recent patents tend to have fewer citations than older ones. In order to ease the comparisons between technology fronts we normalized each front’s FORPAT dividing it by the average FORPAT for each time interval. Data points above one (red dashed line) show receiving higher than average citation during that period (Fig 12), there were no citations received for any of the fronts in year 2017.

The T&P front clearly stands out as the technology front with a higher number of citations per patent for the entire time interval where this front is present. As discussed before, this indicator is considered as a proxy for technological impact. In our context, this could mean that transversal developments in precision mechanical elements are of key importance for the development of 3D printing technology. This is a technological feature directly related to the resolution of the printed object, and a key enabling technology (among others) when printing complex geometries. Furthermore, PRINTHEAD is also cited above average, and it is related with technical features similar to those of the T&P front, particularly considering the maximum printing resolution achievable with the device. According to our data, these two trends would indicate an evolution of technology closely pursuing higher resolution printing techniques, perhaps to the detriment of other improvement areas. The spike in relative citations for LASERBEAM front is also remarkable, a sign that should be further studied as more data become available. On the other side, MULTILAYER front consistently performs below average, pointing at a decrease in the technological impact of layer-by-layer based multimaterial printing technologies, including surface treatments based on 3D technology.

**Fig 13:**
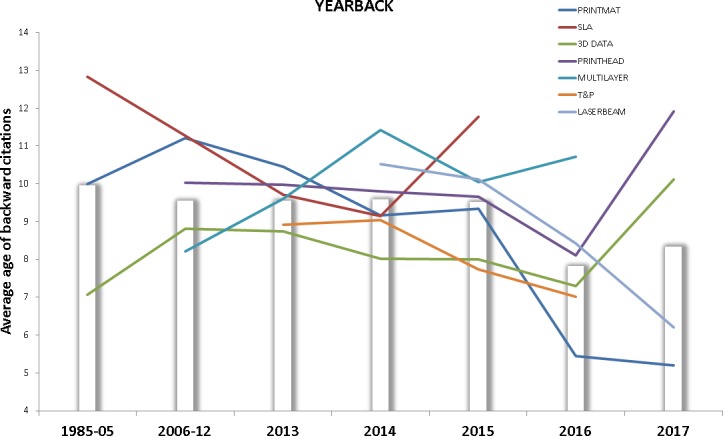
YEARBACK for each technology front. This figure shows the evolution of YEARBACK indicator for each technology front. Data points above the bars indicate older than average backward citations.

An analysis of the average age of the citations made by the patents on each technology front (YEARBACK) is shown in Fig 13, the bars show the average age of citations made in each time period. Values below these bars indicate that the technology front is citing more recent patents than the average corresponding to that time interval.

When patents corresponding to a field tend to cite more recently developed technologies, it is usually interpreted as a sign of increased rate of technology change. T&P technology front cites more recent developments across all the time intervals analyzed, thus complementing the “high relevance” signal returned by this front with FORPAT with an “increased rate of change” indicator, as shown by YEARBACK. 3D DATA, as to be expected from a strongly software-based technology front, remains below average with the exception of a spike in year 2017. On the contrary, MULTILAYER and SLA show relatively old citations when compared to the rest of fronts, pointing at the possibility of stagnation of these fields. Both LASERBEAM and PRINTMAT show a persistent “modernization” of their citation patterns, further research could reveal if this correlation is due to the development of new metal/ceramic materials for laser/electron beam 3D printing.

**Fig 14:**
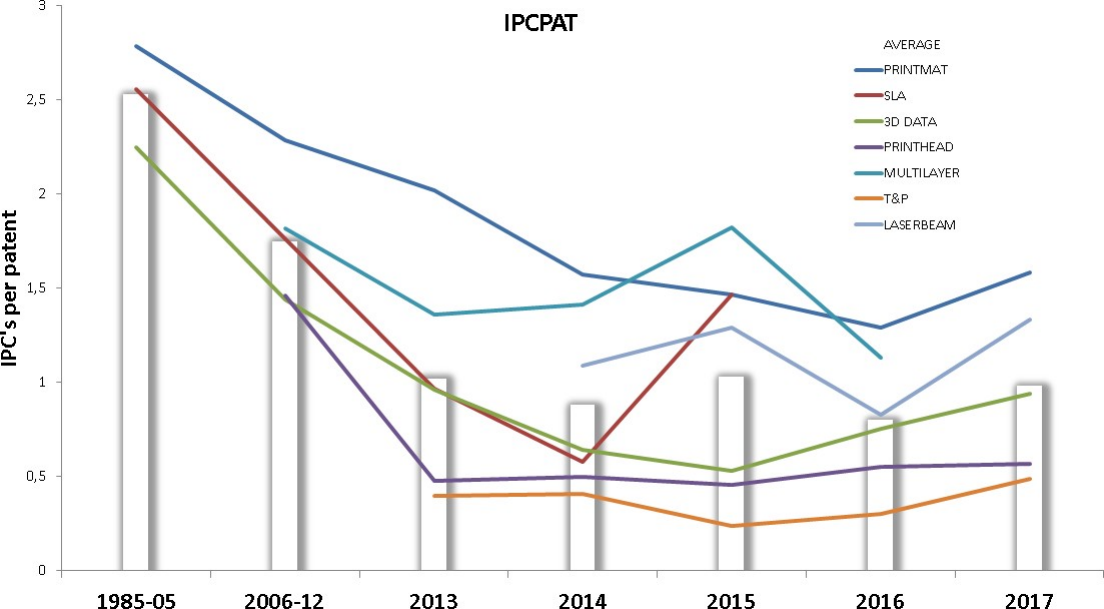
IPCPAT for each technology front. This figure shows the evolution of IPCPAT indicator for each technology front. Data points above the bars indicate higher than average IPC’s per patent rates.

**Fig 15:**
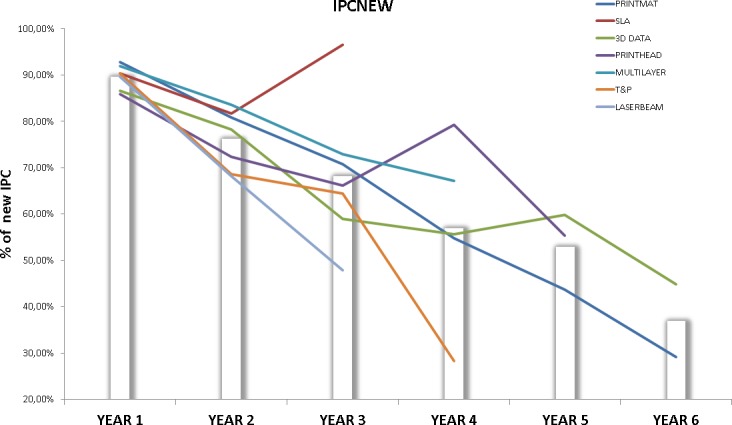
IPCNEW for each technology front. This figure shows the evolution of IPCNEW indicator for each technology front. Data points above the bars indicate higher than average incorporation of new IPC’s to the technology front.

The interdisciplinarity of a technology front is also an interesting factor to analyze, since the biggest opportunities for innovation often emerge from the interaction of different technical fields. Here we base our analysis on two indicators, the number of unique IPC codes per patent (IPCPAT) on one side, and the % of new IPC codes (IPC codes not present before, IPCNEW) that are unique to each time interval on the other. Figs 14 and 15 respectively show the values of these indicators over time, the bars indicate the average value of each indicator for each interval.

The technological diversity indicator IPCPAT behaves as expected: the predominantly mechanical technology fronts (PRINTHEAD, T&P) are well below the average while MULTILAYER and PRINTMAT are above, probably due to the variety of materials and techniques that these technology fronts involve, which increase the total number of unique IPCs present in the technology front. LASERBEAM is also above average across all the intervals, indicating a variety of technologies and materials forming part of this front. Despite being on the average of the rest of technologies, the interdisciplinarity in SLA shows a spike in year 2017 that will be corroborated by other variables detailed further on in this study.

As explained before, it is logical to find a decreasing trend on the IPCNEW indicator in all technologies, given the increased difficulty over time for an IPC to qualify as “new”. However, some interesting conclusions can be drawn from Fig 15.

While IPCPAT gives information about the interdisciplinarity of a given technology, IPCNEW informs about the level of novelty of the diversification taking place in that technology. This helps us to put the increase in multidisciplinarity of LASERBEAM and PRINTMAT into context: Multidisciplinarity is increasing, but it does so mainly by spreading the technology fields (IPC) that were already present in these fronts. According to our data, MULTILAYER is above the average in both multidisciplinarity and the incorporation of technologies from new fields, thus confirming the technological dynamism of this field. The evolution of 3D DATA may evidence an increasing trend of incorporating new fields into its technical base, but more data is needed to support this point. Finally, T&P stands out as the technology front that is clearly below average on both interdisciplinarity and incorporation of new fields, thus proving the highly specialized nature of this front. Note that SLA exhibits the same spike we detected on IPCPAT.

**Fig 16:**
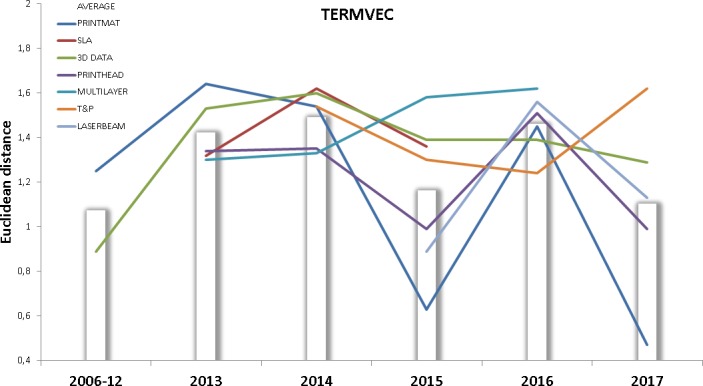
TERMVEC for each technology front. This figure shows the evolution of the TERMVEC indicator for each technology front. Data points above the bars indicate higher than average Euclidean distance between term vectors.

This study also proposes a text mining approach for characterizing the technological change (TERMVEC, NEWTERM). The results of calculating TERMVEC are presented in Fig 16.

The analysis of distances between term vectors sends mixed signals, but we can observe that MULTILAYER is showing certain changes in the concepts it deals with, this observation is coherent with the trend shown by this front in the IPCNEW indicator. If we omit the overall front spike in year 2016 then 3D DATA and T&P are also above the average in term distance. Data points corresponding to year 2017 seem to confirm this behavior, but this result needs to gain consistence in the light of more data. SLA is above average in this indicator, which is coincident—once more—with the signals of technology change sent by previous indicators.

**Fig 17:**
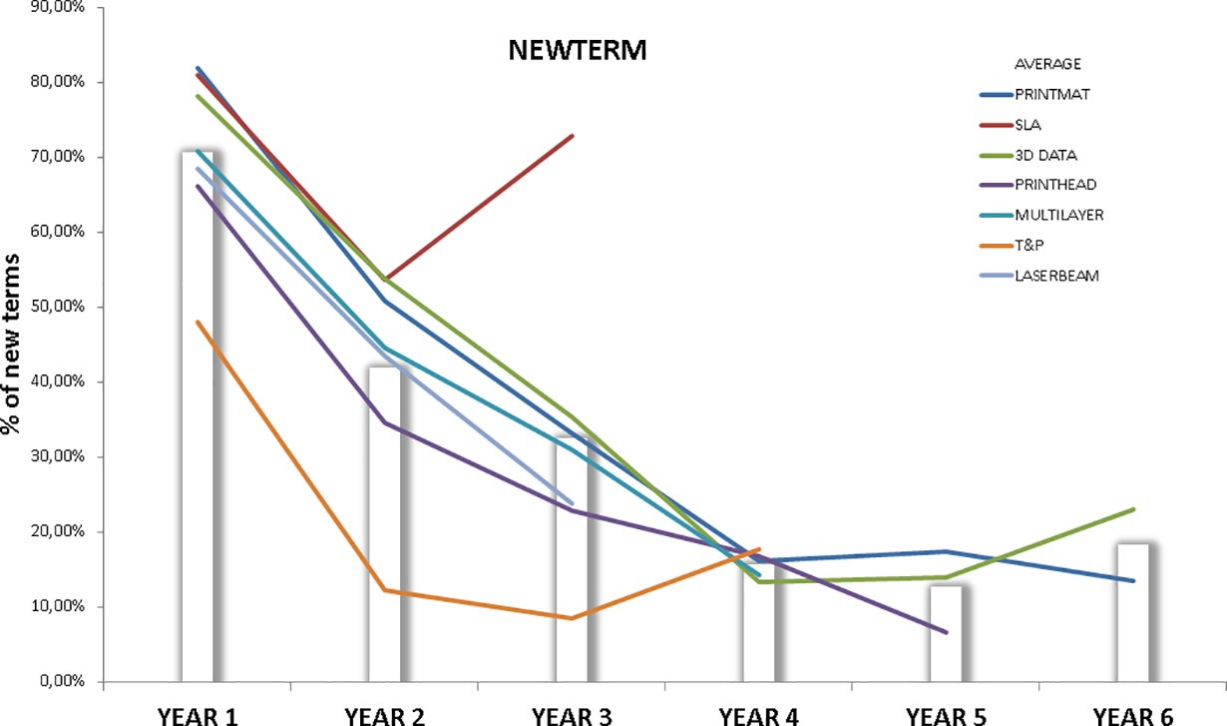
NEWTERM for each technology front. This figure shows the evolution of NEWTERM indicator for each technology front. Data points above the bars indicate higher than average presence of new and relevant terms.

The analysis of NEWTERM (Fig 17) shows that the disparity of concepts unveiled in T&P front is not due to the massive incorporation of new terms. This behavior is also coherent with the “specialization” features of this front revealed in the previous analyses.

The 3D DATA front may tell a different story, given that both text-mining indicators show above the average technology change. PRINTMAT behavior is similar to that of 3D DATA in NEWTERM evolution, but the erratic behavior of the former in TERMVEC indicator makes it difficult to diagnose the changes this field is undergoing. Looking at SLA data, we again get a red flashing light marking a substantial transformation taking place in this field.

## Conclusions

This paper describes our approach for the detection of core technological solutions—which we call “technology fronts”—underlying certain device or broad development (3D printing has been the choice, as explained in the introduction) and the characterization of their dynamics of change across time. After retrieving a dataset containing 3D printing patents from database Patseer, we designed a text-mining procedure that allowed us to identify the most relevant concepts these patents dealt with, according to the statements contained in their claims. These terms were crossed with patents to build term-document matrices corresponding to a set of time intervals that span from year 1985 to 2017. These matrices were analyzed using a topic modeling technique, which has shed light on the technology fronts being developed under the broad field of 3D printing. We found that some of these fronts are in part coincident with the main taxonomies of typical devices in the 3d printing industry, while others describe “hot points” where engineering efforts are put into practice to improve critical aspect of the devices. In order to study the behavior of these technology fronts, and considering the data-features of transversal developments, we opted for a subsetting strategy based on the gamma values returned by the topic modeling solution for each patent, so we could build sub-datasets containing the patents in which claims were clearly focused on the topics identified by our approach. Metrics built on patent data were used to characterize the rate of change of technology fronts, analyzing each of these on a relative basis with respect to the values produced by the rest of the fronts.

The conclusions derived from our work could start by describing the features of the more design related-electromechanical technology fronts underlying 3D printing, identified as T&P (transmission and precision positioning technologies) and PRINTHEAD (design, control and mechanics of printing head) fronts. These fronts show above average technological relevance (FORPAT) and a low multidisciplinary profile, according to almost all indicators. This could also be interpreted in terms of “high specialization” of these technology fronts. Our conclusion is that fast, radical technological transformations are not taking place on these fronts and data does not show evidence of any change in this trend. In spite of this, many other developments may be dependent on the improvement of these technologies, given the vital importance of these elements on the printing resolution capacity, and the increasing demand for geometrically complex, micron-accurate printing of parts. The “enabling” nature of these fronts would therefore explain their relevance.

A very different pattern is found in MULTILAYER. This is a technology front weak in relevance that cites seemingly outdated sources (YEARBACK) but shows very clear signals of undergoing multidisciplinary change, confirmed by both patent and text-mining analyses. We believe that the signals of high dynamism detected on this front are probably related with two of the future priorities for 3D printing described in Table 1, namely the use of multiple printing materials on the object being printed, and the capacity of 3D printers to produce finished products such as more-or-less complete printed circuit boards. According to our data, technology change rate is high on this front and significant future innovations can be expected to come from it. A milder but similar behavior is detected in 3D DATA, text-mining indicators corroborate the trend shown by IPCNEW in the latter stages of this front. In addition to this, the citation patterns also point at a field prone to change. 3D DATA also has a direct impact on the software priorities cited at Table 1 [52].

The analysis of LASERBEAM technology front is conditioned by the scarcity of data available due to its “novelty”: following our method this front is first detected in year 2014, precisely the year in which many laser-sintering patents expired [10,52]. The considerable technical challenges for processing metal and ceramic materials in 3D printing could in part explain this novelty, but the development of this technology front is directly linked with future goals of 3D printing technology such as multimaterial and finished product printing. Our analysis, limited as it is due to the reduced amount of data available, simultaneously points at an increase in relevance and a progressive modernization of the citations (as shown by FORPAT and YEARBACK). This simultaneity indicates that in the future this front may present some of the enabling characteristics that we attributed to PRINTHEAD and T&P in our conclusions. As with these fronts, LASERBEAM shows signs of specialization, despite being more multidisciplinary by nature.

Few conclusions can be extracted from the analysis of PRINTMAT, given the mixed signals produced by our indicators. The highly multidisciplinary nature of this front led us to think that a certain amount of technology changes may come from technical areas that fall beyond 3D printing, therefore not captured by our query.

The SLA front shows very interesting behavior. This is a clearly defined technology front, the interpretation of which did not raise doubts when it was present in an interval, however, its presence is temporarily interrupted in interval 2006–12 and it finally disappears from our model from year 2015 onwards. An analysis of the data shows relatively high values across time in some variables related with technology change (TERMVEC, NEWTERM, IPCNEW), combined with a noticeable spike in year 2015 in IPCPAT, NEWTERM AND IPCNEW. Experts in the field (stereolithography) and our own research suggest that this technology may be undergoing a pivotal change in the dominant technology from ultraviolet light to liquid crystal display based devices, increased speed and reduced cost being some of the advantages of this innovation [82–84]. Such radical changes can significantly alter the vocabulary describing the technology, thus distorting the results of our text-mining based approach, particularly when patents corresponding to the “old-school” technology get mixed in with the patents of the new—noticeably different—technology. We may be at this point regarding SLA technology.

We have presented a reproducible method for studying the underlying technologies that, step by step, advance a device or broad technology (in this case, 3D printing) from early implementations to “hot technologies”, and finally to widespread adoption. The technology fronts and data revealed by our method have been convincing to experts and coincident with many technology trends already pointed out in technical reports and scientific literature. The limitations of our approach include those inherent to every text mining study: the extraordinary amount of noise present in data and the influence that a moderate number of observations can have on the results produced by the method, as shown in the SLA case.

The authors would like to finish this section with the following sincere and perhaps slightly disappointing sentence stated by Jaffe and Fogarty [76]: “Many of the important concepts in the economics of technological change are fundamentally unobservable”. This sentence is rigorously true, and we think that it could not be otherwise, given the extremely multivariate nature of technological change, and the inherent immensurability—incidentally admitting that there are certainly several “unknown unknowns” involved—of many of these variables. We would like to instill the above stated conclusions with a humble admission of this fact.
